# Comparison of Coconut Coir, Rockwool, and Peat Cultivations for Tomato Production: Nutrient Balance, Plant Growth and Fruit Quality

**DOI:** 10.3389/fpls.2017.01327

**Published:** 2017-08-02

**Authors:** Jing Xiong, Yongqiang Tian, Jingguo Wang, Wei Liu, Qing Chen

**Affiliations:** ^1^College of Resources and Environmental Sciences, China Agricultural University Beijing, China; ^2^Vegetable Research Center, Beijing Academy of Agriculture and Forestry Sciences Beijing, China; ^3^College of Horticulture, China Agricultural University Beijing, China

**Keywords:** rockwool, coconut coir, peat, nutrients, plant growth, tomato

## Abstract

Rockwool (RC) and peat are two common substrates used worldwide in horticultural crop production. In recent years environmental and ecological concerns raised the demand for reducing the use of RC and peat. Although coconut coir (CC) has been increasingly used as an alternative to RC and peat, it is still needed to comprehensively evaluate the feasibility of CC before widely used. To meet this need, CC, RC, and peat-vermiculite (PVC) cultivations were used as tomato cultivation substrates to evaluate their effects on EC, pH and mineral ions in root-zone solution and drainage, nutrient uptake by crops, nutrient balance of cultivation system, plant growth and fruit quality. In general, CC significantly increased K and S uptake by crops, photosynthesis, individual fruit weight and total fruit yield compared to RC, and increased P and K uptake by crops and total fruit yield compared to PVC. Moreover, CC significantly increased organic acid of fruit in first truss compared to both RC and PVC. The uncredited nutrient was overally lower under CC than under RC and PVC (the lower, the better). For all substrates, the blossom-end rot (BER) of fruit increased gradually from 3rd to 13th trusses. The BER of fruit was not significantly influenced by CC compared to RC or PVC, but was sginificantly decreased by PVC compared to RC. Our results infer that CC was a potential substrate that could be widely used in tomato production. However, the inhibition of BER was still a challenge when CC was used as cultivation substrate for tomato.

## Introduction

Solid substrate cultivation is common in horticultural crop production around the world, especially for fruity vegetables such as tomato and cucumber. It has been estimated that approximately 95% of greenhouse vegetables are produced using solid substrates in Europe, the United States and Canada (Grunert et al., 2016). Traditionally, rockwool (RC) and peat are two major common materials used in solid substrate cultivation (Bunt, 1988; Sonneveld, 1993; Raviv and Lieth, 2008). RC is mainly made of diabase and limestone through melting at a high temperature (∼1600°C). This material is general suitable for crop gowth due to its stable structure, high water holding capacity, and moderate porosity (Sonneveld, 1993; Raviv and Lieth, 2008). However, since RC is an inorganic material that is hard to degrade, the RC waste is often stockpiled or landfilled, resulting in potential environmental risk (Cheng et al., 2011).

In addition to RC, peat is also used extensively as a cultivation substrate in horticulture because of its desirable physicochemical and biological properties for plant growth (Schmilewski, 2008; Krucker et al., 2010). It was estimated that about 40 million m^3^ of peat is used annually worldwide in horticultural production (Kuisma et al., 2014). Unlike RC, peat is an organic material that can be easily recycled and reused (Gruda, 2012; Raviv, 2013). However, in recent years environmental and ecological concerns raised the demand for reducing the use of peat because its harvest is destroying endangered wetland ecosystems worldwide (Steiner and Harttung, 2014).

Since both RC and peat have their own limitations, coconut coir (CC), an environmental friendly material with stable physicochemical and biological properties, has been increasingly used as a cultivation substrate in horticultural production (Barrett et al., 2016). CC is the coconut waste consisting of the dust and short fibers and approximately 12 million tones are produced annually in the world (Nichols, 2013). Due to its good water retention and aeration characteristics, CC has gradually become the most potential alternative to both RC and peat in substrate cultivation. Therefore, it is necessary and important to evaluate the efficiency of CC when widely used in crop production.

In substrate cultivation, crops were planted in a small volume of growing media, resulting in limited nutrients and water for root absorption. Hence, mineral nutrient management is a key factor determining the yield and nutritional quality of vegetable crops during substrate cultivation (Kader, 2008; Fallovo et al., 2009). Generally, the retention, movement and availability of mineral nutrients in root-zone are related to several properties of a substrate, such as particle size, water and nutrient holding capacities, cation exchange capacity and nutrient content (Ao et al., 2008; Urrestarazu et al., 2008; Carmona et al., 2012; Asaduzzaman et al., 2013). Therefore, to match nutrient requirements of crops, the adjustment of mineral nutrient contents in the supplied nutrient solution should be considered based on substrate properties. CC, peat, and RC often have different physicochemical properties. For instance, CC has higher P, K, Na, and Cl contents compared to peat, and lower porosity and water-holding capacity compared to RC (Abad et al., 2002; Mazuela, 2005). Those difference can affect the nutrient management during the cultivation. Hence, it is necessary and important to evaluate the available nutrient contents in root-zone solution of different substrates.

Tomato is one of most economically important vegetable crops in the world. During greenhouse production, tomato is mainly produced using RC and peat as cultivation substrates. Although CC has been increasingly used as an alternative to RC and peat in greenhouse tomato production, little information is available regarding the difference among these substrates in the retention, movement and availability of mineral nutrients in root-zone. The objectives of this study were to investigate the effects of RC, peat, and CC on root-zone nutrient retention and movement, nutrient balance, plant growth and tomato fruit quality, and to explore the major factor influencing the adjustment of mineral nutrients in the supplied nutrient solution.

## Materials and Methods

### Experimental Site and Crop Planting

The experiment was conducted in a climate-controlled greenhouse at the Beijing Vegetable Research Center, Beijing Academy of Agriculture and Forestry Sciences in Beijing from 11 October 2014 to 26 May 2015. The average light intensity ranged from 18.3 to 136.8 μmol m^-2^ s^-1^, and the average temperature ranged from 14.0 to 23.0°C, respectively.

Tomato (*Lycopersicon esculentum* Mill. *Lucius* F1) seeds were sown on 1 September 2014 and transplanted to substrate cubes (10 cm × 10 cm) on 22 September 2014. Eighteen days after planting on the substrate cube, tomato crops were transplanted to substrate slabs (100 cm × 20 cm × 7.5 cm) with 30-cm plant spacing. The planting density was 2.4 crops m^-2^.

### Experimental Design

The following substrates including RC, CC and the mixture of peat and vermiculite (*v*/*v*, 2:1) (PVC) were used as cultivation substrates in the experiment. RC and CC were bought from Grodan Group and Jiffy Group in Netherland, respectively. Both peat and vermiculite were bought from Beijing Lide Agricultural S&T Development Company in China. Selected characteristics of different substrates were showed in **Table 1**. The experiment was a completely randomized block design with three replicates and each replicate contained one cultivation gutter (1000 cm × 32 cm × 10 cm). For each cultivation gutter, 10 substrate slabs were installed.

**Table 1:** Selected physical and chemical properties of rockwool, coir, and peat-vermiculite.

Properties	Rockwool	Coir	Peat-vermiculite
EC (dS m^-1^)	0.06	0.10	1.10
pH	6.5	6.1	7.1
C (%)	2.2	49.5	15.9
N (mg kg^-1^)	56	44	64
P (mg kg^-1^)	30	38	42
K (mg kg^-1^)	178	1560	246
Ca (mg kg^-1^)	279	58	1668
Mg (mg kg^-1^)	216	55	636
S (mg kg^-1^)	303	405	645
Porosity (%)	89.2	85.6	66.0
Water porosity (%)	84.1	80.0	64.0
Air porosity (%)	5.1	5.6	2.0
Bulk density (g cm^-3^)	0.08	0.2	0.4

### Nutrient Solution Management

Compositions of standard nutrient solution were 15.4 mmol L^-1^ NO_3_^-^, 1.4 mmol L^-1^ NH_4_^+^, 1.8 mmol L^-1^ H_2_PO_4_^-^, 9.3 mmol L^-1^ K^+^, 3.9 mmol L^-1^ Ca^2+^, 1.4 mmol L^-1^ Mg^2+^, 2.1 mmol L^-1^ SO_4_^2-^, 14.7 μmol L^-1^ Fe, 27.8 μmol L^-1^ Mn, 0.8 μmol L^-1^ Cu, 6.7 μmol L^-1^ Zn, 4.20 μmol L^-1^ B, and 0.07 μmol L^-1^ Mo. The NO_3_^-^/NH_4_^+^ and K^+^/Ca^2+^ ratios were 11 and 2.36, respectively. The electrical conductivity (EC) and pH in reservoir tanks were monitored every week using a multi meter (Multi 3420 SET C., WTW, Germany). To maintain the set EC of 2.3 dS m^-1^, fresh water (EC 0.12 dS m^-1^, pH 7.18, Na^+^ 0.6 mmol L^-1^, Ca^2+^0.1 mmol L^-1^, Mg^2+^ 0.05 mmol L^-1^, SO_4_^2-^ 0.2 mmol L^-1^, NO_3_^-^ 0.7 mmol L^-1^, NH_4_^+^ 0.05 mmol L^-1^ and H_2_PO_4_^-^ 0.02 mmol L^-1^) and fresh nutrient solution were added to the tank to reach the fixed volume (200 L) of nutrient solution. The irrigation system was closed system. Each gutter had one reservoir tank. The drainage reached directly the reservoir tank where it was mixed with the new solution.

The nutrient solution was applied through a drip (average flow rate of 1.5 L h^-1^) irrigation system with one dipper per plant. Drainage ratio was maintained within 20–50% at each irrigation event. The irrigation frequency and volume were the same for all cultivation gutters. During the first 8-week period, nutrient solution was supplied for two times per day (9:00 and 13:00) for 20 min each, irrigation volume was 1 L per plant. During the next 25-week period, nutrient solution was supplied for four times per day (9:00, 11:00, 13:00, and 15:00) for 20 min each, irrigation volume was 2 L per plant. Every 2 months, the nutrient solution tank was washed and the nutrient solution in the tank was thrown away.

### Root-Zone Solution and Drainage Analysis

From 4 weeks after transplanting, root-zone solution and drainage were sampled every 2 or 3 weeks. Root-zone solution (100 ml) was collected with a root solution extractor installed between the crops, while drainage (100 ml) was collected from the drainage tank. The samples were stored at 2°C until further analyzing. The EC and pH were measured by using a multi meter (Multi 3420 SET C., WTW, Germany). NO_3_^-^ was assayed by a continuous flowing analyzer (AA3, Seal, Germany). K^+^, Ca^2+^, Mg^2+^, and H_2_PO_4_^-^ were assayed by inductively coupled plasma spectrometry (ICPE-9000, Shimazu, Janpan). SO_4_^2-^ was assayed by inductively coupled plasma spectrometry (ICP-MS 7900, Agilent Technologies, United States).

### Plant Nutrient Analysis

On weeks 3, 6, 10, 16, 25, and 33 after transplanting, stems, leaves and fruits were sampled, washed with distilled water, and then dried in a ventilated oven at 75°C to constant weight. Nutrient contents in leaves and fruits samples were analyzed. The contents of K, Ca, Mg, and P were assayed after digestion with H_2_SO_4_-HNO_3_-HClO_4_ (H_2_SO_4_:HNO_3_:HClO_4_ = 1 ml:5 ml:1 ml) by inductively coupled plasma spectrometry (ICPE-9000, Shimazu, Japan; ICP-MS 7900, Agilent Technologies, United States). The N content was assayed after digestion with H_2_SO_4_-H_2_O_2_ by continuous flowing analyzer (AA3, Seal, Germany). The S content was assayed after digestion with HNO_3_ by inductively coupled plasma spectrometry (ICP-MS 7900, Agilent Technologies, United States) (Zhou et al., 2000).

### Malondialdehyde, Antioxidative Enzymes and Photosynthesis in Leaves

On day 207 after the transplanting, the malondialdehyde (MDA), superoxide dismutase (SOD), catalase (CAT) and peroxidase (POD) in leaves were measured as the methods described in Gao (2006). In addition, the photosynthetic rate (Pn), stomatal conductance (Gs), intercellular CO_2_ concentration (Ci) and evaporation rate (E) of a fully developed leaf was also measured using a LI-6400 portable photosynthesis system (LI-COR Inc., Lincoln, NE, United States).

### Fruit Yield and Quality

During fruit ripening period, for each cultivation gutter, fruits were harvested from 24 crops to measure the individual fruit weight, fruit number and fresh yield. Individual fruit weight was measured using electronic balance. At the end of the cropping season, the fresh yield of each harvest was summed up as the total yield (Y). The total number of fruits and the number of fruits affected by blossom-end rot (BER) were determined at each harvest time. The black tissue at the end of fruit is the incidence of BER. Moreover, 1.5 kg ripe fruits were sampled from each cultivation gutter to measure soluble solids, reducing sugars, organic acids, and vitamin C (Li, 2010).

### Nutrient Balance

Nutrient balance was calculated in different substrate cultivations. When prepare the fresh nutrient solution, nutrient inputs was recorded. Nutrient solution was sampled when clean the nutrient solution tank. At the end of the trial, the substrate was sampled. Nutrient contents were analyzed as methods described in “Discussion.” The uncredited nutrient was calculated as follows:

Uncredited nutrient = Nutrient input – Nutrient uptake by crops – N residues in substrate.

### Statistical Analysis

Data were subjected to an analysis of variance (ANOVA) using SPSS 20.0 software (SPSS statistical package, Chicago, IL, United States). The statistical significance of the results was analyzed by the LSD test at the 0.05 level.

## Results

### EC and pH in Root-Zone Solution and Drainage

**FIGURE 1:**
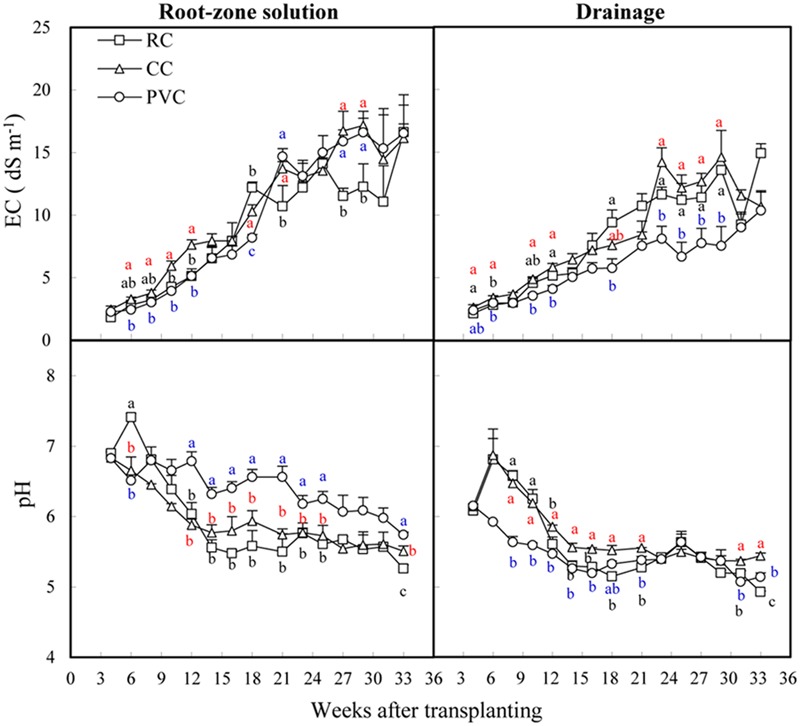
Electrical conductivity (EC) and pH in root-zone solution and drainage under rockwool (RC), coir (CC) and peat-vermiculite (PVC) cultivations. The vertical bars represent the standard errors. Different letters indicate significant difference between treatments according LSD test at *P* < 0.05. Black letter, red letter and blue letter denote rockwool (RC), coir (CC) and PVC cultivations respectively.

The EC in both root-zone solution and drainage of all substrates increased gradually during the first 21-week period after transplanting and were then maintained at relatively stable levels during the next 9 weeks (**Figure 1**). In general, EC in drainage was lower in PVC than in RC and CC.

In contrast to EC, pH in both root-zone solution and drainage of RC and CC decreased gradually during the first 14-week period after transplanting and then maintained at relatively stable levels during the next 19 weeks. Under PVC, pH decreased slowly during the first 23-week period after transplanting. During the growing period, the fluctuation of pH in both root-zone solution and drainage were lower in PVC than in RC and CC. Overally, PVC showed higher pH in both root-zone solution at most sampling times but lower pH in drainage from weeks 6 to 16 after transplanting.

### Ions Dynamic in Root-Zone Solution and Drainage

**FIGURE 2:**
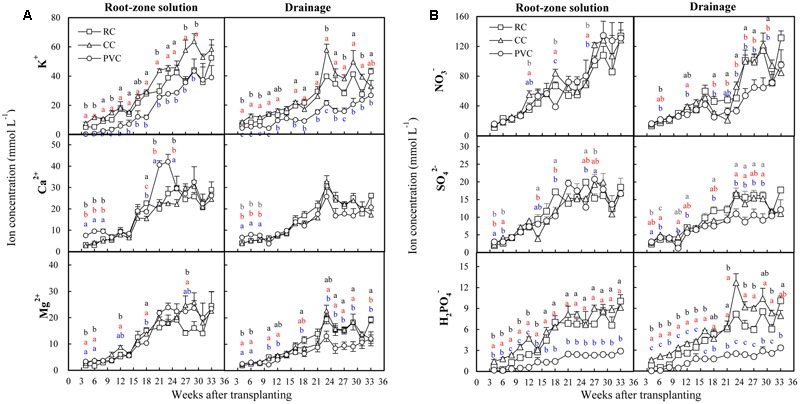
Cations **(A)** and anions **(B)** in root-zone solution and drainage under RC, CC and PVC cultivations. The vertical bars represent the standard errors. Different letters indicate significant difference between treatments according LSD test at *P* < 0.05. Black letter, red letter, and blue letter denote RC, CC and PVC cultivations respectively.

The concentrations of K^+^ in both root-zone solution and drainage of all substrates increased gradually during the growing period, and were generally lower in PVC than in RC and CC (**Figure 2A**). Moreover, CC showed the highest K^+^ concentration in both root-zone solution and drainage at most sampling times. The concentrations of Ca^2+^ and Mg^2+^ in both root-zone solution and drainage increased gradually during the first 23-week period after transplanting and were then maintained at relatively stable levels during the next 10 weeks (**Figure 2A**). In general, the PVC showed higher Ca^2+^ concentration in root-zone solution on weeks 4, 6, 8, 18, 21, and 23 after transplanting, but showed lower Mg^2+^ concentration in drainage from weeks 8 to 31 after transplanting, when compared to RC and CC.

The concentrations of both NO_3_^-^ and SO_4_^2-^ in root-zone solution increased gradually during the growing period and were not influenced by substrates (**Figure 2B**). However, the NO_3_^-^ and SO_4_^2-^ in drainage were significantly influenced by substrates. Among substrates, RC showed higher NO_3_^-^ and SO_4_^2-^ in drainage from weeks 16 to 21 after transplanting, while PVC showed lower NO_3_^-^ and SO_4_^2-^ in drainage from weeks 23 to 29 after transplanting. The concentrations of H_2_PO_4_^-^ in both root-zone solution and drainage were significantly influenced by substrates and were obviously lower in PVC than in RC and CC. Moreover, CC showed the highest H_2_PO_4_^-^ in both root-zone solution and drainage at most sampling times.

### Ratios between Different Ions in Root-Zone Solution

The K^+^/Ca^2+^, Mg^2+^/Ca^2+^, K^+^/Mg^2+^, and Ca^2+^/H_2_PO_4_^-^ ratios in root-zone solution were all significantly influenced by substrates (Supplementary Figure S1). In general, during the whole growing period, CC showed the highest while PVC showed the lowest K^+^/Ca^2+^ ratio in root-zone solution. The average K^+^/Ca^2+^ ratios in root-zone solutions of RC, CC and PVC were 1.6, 2.3, and 0.8, respectively. It was noted that the K^+^/Ca^2+^ ratio of CC fluctuated around that of nutrient solution (2.3). For all substrates the Mg^2+^/Ca^2+^ ratios in root-zone solution were obviously higher than that of nutrient solution (0.4). CC showed higher Mg^2+^/Ca^2+^ ratio from weeks 4 to 18 after transplanting, when compared to RC and PVC. In contrast to the Mg^2+^/Ca^2+^ ratio, for all substrates the K^+^/Mg^2+^ ratio in root-zone solution was lower than that of nutrient solution (6.5). During the whole growing period, PVC showed the lowest K^+^/Mg^2+^ ratio in root-zone solution. A reverse trend was found in the Ca^2+^/H_2_PO_4_^-^ ratio. No obvious differences were found between RC and CC in both K^+^/Mg^2+^ and Ca^2+^/H_2_PO_4_^-^ ratios.

### Biomass, Nutrient Concentration and Uptake in Crops

**FIGURE 3:**
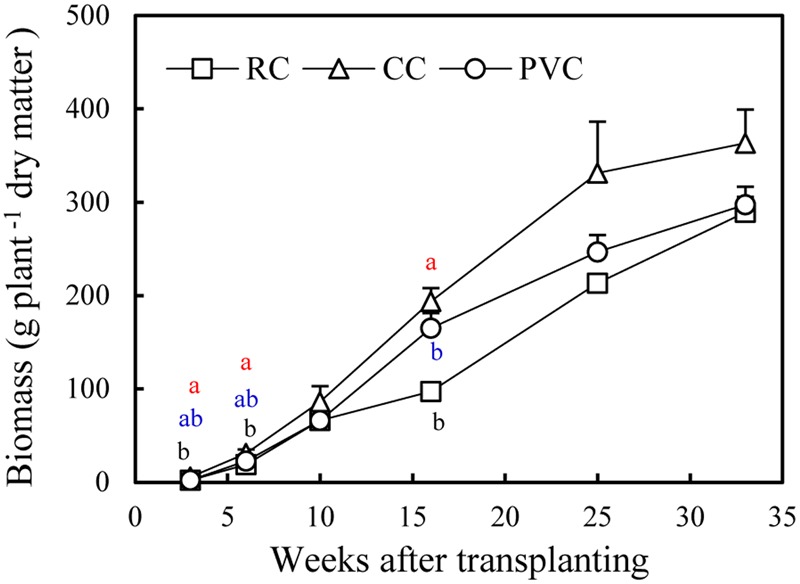
Biomass of crop under RC, CC, and PVC cultivations. The vertical bars represent the standard errors. Different letters indicate significant difference between treatments according LSD test at *P* < 0.05. Black letter, red letter and blue letter denote RC, CC and PVC cultivations respectively.

Substrates influenced plant biomass (**Figure 3**). In general, CC had the highest biomass while RC had the lowest.

**FIGURE 4:**
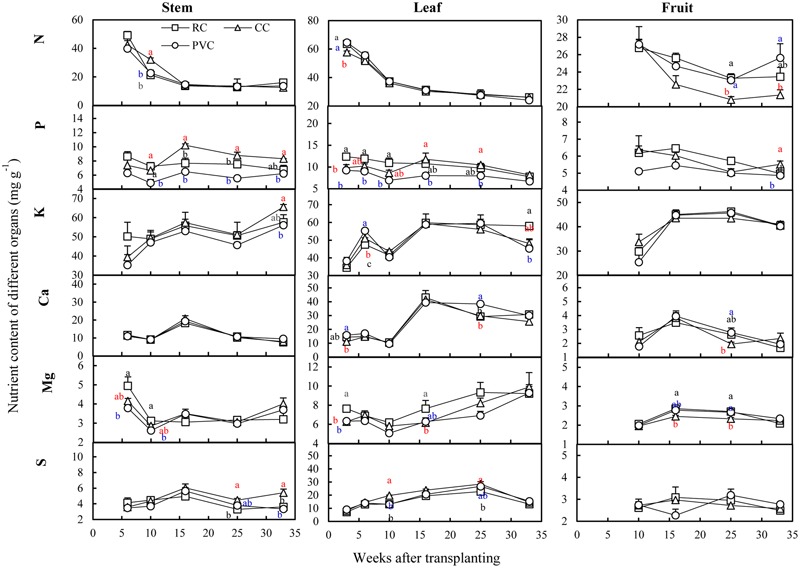
Nutrient concentration in crop under RC, CC, and PVC cultivations. The vertical bars represent the standard errors. Different letters indicate significant difference between treatments according LSD test at *P* < 0.05. Black letter, red letter and blue letter denote rockwool (RC), coir (CC) and peat-vermiculite (PVC) cultivations respectively.

Substrates did not statistically influence the concentrations of N, K, Ca, Mg, and S in stem, leaf and fruit of tomato, but significantly influenced P concentrations (**Figure 4**). Overally, PVC showed lower P concentrations in stem, leaf and fruit compared to RC and CC, and CC showed higher P concentrations in stem compared to RC.

Substrates significantly influenced the accumulation of N, P, K, and S nutrient in crops (Supplementary Figure S2). In general, all nutrients showed the highest accumulation in crops under CC but the lowest accumulation in crops under RC.

### Nutrient Balance of Different Substrate Cultivations

Although no significant difference was found in nutrient input among different substrate cultivations, different substrate cultivations showed significant differences in nutrient uptake by crops and nutrient residue in substrates, resulting in obvious differences in nutrient balance (**Table 2**). The CC cultivation generally showed the highest nutrient uptake by crops, especially for P, K, and S. Moreover, the CC cultivation also showed the highest P residue in substrate. However, the highest residues in substrate of other nutrients (e.g., Ca, Mg, and S) were generally found in the PVC cultivation. Due to these differences, CC generally showed the lowest uncredited nutrient (the lower, the better), especially for N, P, and K. In addition, the lowest uncredited Ca was found under the PVC cultivation, and both CC and PVC showed lower uncredited Mg and S compared to RC.

**Table 2:** Nutrient balance under rockwool (RC), coir (CC), and peat-vermiculite (PVC) cultivations.

Nutrients (g m^-2^)	Substrates	N	P	K	Ca	Mg	S
Input	RC	60.0 a^a^	12.3 a	88.3 a	34.4 a	6.4 a	16.8 a
	CC	56.1 a	12.1 a	84.9 a	34.9 a	6.9 a	15.9 a
	PVC	59.2 a	13.3 a	91.2 a	36.2 a	7.4 a	16.9 a
Uptake by crops	RC	15.9 a	4.3 ab	34.0 b	8.0 a	3.6 a	4.1 b
	CC	18.7 a	6.0 a	41.2 a	9.5 a	3.7 a	6.1 a
	PVC	16.4 a	4.1 b	31.9 b	8.5 a	3.4 a	4.9 ab
Residues in substrate	RC	1.6 b	0.2 b	4.7 b	6.2 c	2.3 b	0.6 c
	CC	7.8 a	1.2 a	17.2 a	18.3 b	3.1 ab	8.0 b
	PVC	8.2 a	0.2 b	17.4 a	23.6 a	3.97 a	10.5 a
Uncredited nutrient	RC	42.5 a	7.8 a	49.6 a	20.2 a	0.5 a	12.1 a
	CC	29.6 b	4.9 b	26.5 b	7.1 b	0.1 b	1.8 b
	PVC	34.6 ab	9.0 a	41.9 a	4.1 c	0.03 b	1.5 b

*^a^The same letter denotes no significant difference among different substrates (*P* = 0.05).*

### Photosynthesis, Malondialdehyde and Antioxidative Enzymes in Leaves

All photosynthesis-related parameters (Pn, Gs, Ci, and E) were significantly higher under CC and PVC than under RC, and no significant difference was found between CC and PVC (Supplementary Table S1). However, there was no significant difference in MDA, SOD, POD, and CAT among all substrate cultivations.

### Yield, Blossom-End Rot and Quality of Fruits

The individual fruit weight was generally higher under CC and PVC than under RC, especially for the 6th and 7th trusses (**Table 3**). No significant difference was found in the average of individual fruit weight between CC and PVC. However, since CC had significantly higher fruit yield in the 5th, 7th, and 8–13th trusses, the total fruit yield was significantly higher under CC than PVC. In addition, both CC and PVC had significantly higher total fruit yield compared to RC. For most lower trusses (e.g., 1st, 2nd, and 4–7th), the BER was not influenced by substrates. However, for the 3rd and higher trusses (8–13th), the BER was significantly higher under RC and under PVC. Effects of substrates on fruit quality were generally not obvious, and only for first truss a significant higher organic acid was found under CC compared to RC and PVC (Supplementary Table S2).

**Table 3:** Individual fruit weight, fruit yield and blossom-end rot under rockwool (RC), coir (CC), and peat-vermiculite (PVC) cultivations.

Parameters	Treatments	1st truss	2nd truss	3rd truss	4th truss	5th truss	6th truss	7th truss	8–13th truss	Average	Total
Individual fruit weight (g)	RC	86.8 a^a^	85.9 a	79.0 b	71.3 a	61.5 b	59.5 b	50.5 b	40.0 b	66.8 b	-
	CC	85.6 a	83.6 a	77.8 b	79.3 a	75.3 a	69.3 a	70.9 a	50.1 a	74.0 a	-
	PVC	90.7 a	92.4 a	99.6 a	79.2 a	68.6 ab	65.6 a	61.1 a	43.3 ab	75.1 a	-
Fruit yield (t hm^-2^)	RC	8.6 a	7.8 b	9.2 a	6.7 b	4.7 b	4.9 a	3.6 b	21.9 c	-	67.5 c
	CC	8.3 a	9.9 a	7.9 a	8.0 a	6.8 a	5.2 a	5.3 a	33.8 a	-	84.9 a
	PVC	8.1 a	9.5 ab	10.3 a	6.9 ab	5.4 b	4.9 a	4.0 b	27.6 b	-	76.9 b
Blossom-end rot (%)	RC	0	0	0.3 a	3.1 a	2.6 a	4.9 a	9.2 a	42.8 a	-	21.2 a
	CC	0	0	2.3 a	2.1 a	1.5 a	4.9 a	8.1 a	36.6 ab	-	19.5 ab
	PVC	0	0	0 b	1.3 a	1.3 a	3.9 a	8.7 a	31.0 b	-	16.9 b

*^a^The same letter denotes no significant difference among different substrates (*P* = 0.05).*

## Discussion

During substrate cultivation, traditionally used RC and peat have their own limitations because of environmental and ecological impacts (Cheng et al., 2011; Steiner and Harttung, 2014). Although CC has been increasingly used as an alternative to RC and peat, it is still needed to fully compare and evaluate the difference among different substrates before widely used in crop production.

Mineral ions and EC in root-zone are critical for plant growth. For all substrates, most mineral ions increased gradually as the growing time increased (**Figure 2**), resulting in gradually increased EC in root-zone (**Figure 1**). In root-zone K^+^, Ca^2+^, and H_2_PO_4_^-^ were the major mineral ions influenced by substrates (**Figure 2**). Although both CC and PVC are organic substrates, the average K^+^ concentration in root-zone was increased by CC but decreased by PVC, when compared to the inorganic RC. This could be due to that CC released K^+^ to solution (Schmilewski, 2008; Barrett et al., 2016), while peat adsorbed K^+^ due to its high cation exchange capacity (Rippy and Nelson, 2007). Potassium is required in the largest amount by tomato crops and is a major elements in determining fruit quality of tomato (Schwarz et al., 2013). The relatively higher K^+^ in root-zone solution under CC (**Figure 2A**) suggested that CC had a high potential to enhance tomato growth. Indeed, the K content in substrate (**Table 1**), the K accumulation in crops (Supplementary Figure S2) and fruit yield (**Table 3**) were significantly higher under CC than under RC and PVC. However, the K-Ca and K-Mg antagonisms are common phenomenon in tomato production (Kabu and Toop, 1970; Pujos and Morard, 1997). Thus, the relatively high K in CC (**Table 1**) might induce Ca and Mg deficiency in crops. Indeed, the K^+^/Ca^2+^ and K^+^/Mg^2+^ ratios in root-zone solution were generally high under CC (Supplementary Figure S1). However, both K-Ca and K-Mg antagonisms were not observed under CC cultivation because Ca and Mg concentrations in stem, leaf and fruit were not influenced by substrates (**Figure 4**), and because the accumulated Ca and Mg in crops was relatively higher under CC than under RC and PVC (Supplementary Figure S2).

The concentration of Ca^2+^ in root-zone solution was increased by PVC compared to RC and CC in early period (before 10 weeks after transplanting; **Figure 2A**). This could be due to that exchangeable Ca^2+^ accounted for the highest proportion (approximately 57.2–82.1%) of the total exchangeable bases of peat (Rippy and Nelson, 2007), leading to the high release of Ca^2+^ from peat to root-zone solution. However, for all substrates the Ca^2+^ concentration in root-zone solution increased gradually as the growing time increased (**Figure 2A**). This is probably due to the gradually decreased pH in root-zone solution during the growing period (**Figure 1**). Low pH could facilitate resolution of Ca^2+^, which might further increase Ca^2+^ contents in root-zone solution (Mao et al., 2005). Significant difference of Ca^2+^ in root-zone solution resulted in different Ca accumulation in crops among treatments (Supplementary Figure S2). It is well-known that Ca deficiency could lead to BER in tomato (De Freitas et al., 2011; Uozumi et al., 2012). Since both CC and PVC showed relatively higher Ca accumulation in crops (Supplementary Figure S2) but lower BER (**Table 3**), the organic substrates might be more efficient than inorganic substrate (RC) in reducing Ca deficiency and BER.

The concentration of H_2_PO_4_^-^ in root-zone solution was obviously lower under PVC than under RC and CC (**Figure 2B**). One reason is that peat adsorbed H_2_PO_4_^-^ due to its high cation exchange capacity (Rippy and Nelson, 2007). Another reason is probably due to that high Ca in peat (**Table 1**) could combine with H_2_PO_4_^-^ to reduce water-soluble H_2_PO_4_^-^ content (Kruse et al., 2015; Cerozi and Fitzsimmons, 2016). Indeed, the Ca^2+^/H_2_PO_4_^-^ ratio in root-zone solution was obviously higher under PVC than under RC and CC during the whole growing period (Supplementary Figure S1). Although no obvious difference in the H_2_PO_4_^-^ concentration in root-zone solution was observed between RC and CC (**Figure 2B**), P accumulation in crops was significantly lower under RC than under CC (Supplementary Figure S2). Since the photosynthetic rate (Pn), stomatal conductance (Gs), intercellular CO_2_ concentration (Ci) and evaporation rate (E) in leaves were all significantly decreased by RC compared to CC (Supplementary Table S1), the decreased photosynthesis might limit P uptake by crops under RC cultivation.

High EC may inhibit nutrient absorption by crops and lead to yield reduction (Rodríguez-Delfína et al., 2012). In tomato production, high-EC induced inhibition of Ca absorption is very common in substrate cultivation, which often leads to BER of tomatoes due to Ca deficiency (Uozumi et al., 2012). In this study, as EC in root-zone solution increased gradually during the growing period (**Figure 1**), BER increased gradually for all substrates from 3rd to 13th trusses (**Table 3**), indicating the Ca deficiency induced by high EC (Neocleous and Savvas, 2015). This result suggested that inhibition of Ca deficiency was still a challenge for soilless tomato production. Despite this, PVC cultivation generally showed the lowest BER (**Table 3**). This phenomenon could be explained by the fact that (1) peat contained high content of Ca (**Table 1**) and was able to enhance Ca absorption by tomato crops (Zhang et al., 2015), (2) lower K^+^/Ca^2+^ ratio in root-zone solution under PVC (Supplementary Figure S1) reduced K-Ca antagonism in root-zone (Neocleous and Savvas, 2015) and (3) the relatively high buffer ability of peat-vermiculite (PVC) resulted in a relatively stable pH during the growing period (**Figure 1**) and benefited Ca uptake by tomato crops (Rippy, 2005). Despite the benefits of PVC, no statistical difference in total BER was found between CC and PVC (**Table 3**). Moreover, CC had significantly higher total fruit yield compared to PVC (**Table 3**), because of the higher nutrient uptake by crops (**Table 2** and Supplementary Figure S2). The advantages of CC also were also reflected in the lower uncredited P and K (the lower, the better; **Table 2**) and higher organic acid in fruit of first truss compared to PVC (Supplementary Table S2).

## Conclusion

Coconut coir was a potential substrate that could be widely used in tomato production. Compared with RC, CC showed higher K and S uptake by crops, photosynthesis, individual fruit weight and total fruit yield, and lower uncredited nutrient (the lower, the better). Compared with PVC, CC showed higher P and K uptake by crops and total fruit yield, and lower uncredited P and K. CC did not influence BER compared to RC or PVC. In addition, effects of substrates on fruit quality were generally not obvious.

## Author Contributions

JX: substantial contributions to the design of the work. Substantial contributions to the acquisition, analysis, interpretation of data for the work. YT: drafting the work or revising it critically for important intellectual content. JW: drafting the work or revising it critically for important intellectual content. WL: agreement to be accountable for all aspects of the work in ensuring that questions related to the accuracy or integrity of any part of the work are appropriately investigated and resolved. Final approval of the version to be published. QC: agreement to be accountable for all aspects of the work in ensuring that questions related to the accuracy or integrity of any part of the work are appropriately investigated and resolved. Final approval of the version to be published.

## Conflict of Interest Statement

The authors declare that the research was conducted in the absence of any commercial or financial relationships that could be construed as a potential conflict of interest.

## References

[B1] Abad M. Noguera P. Puchades R. Maquieira A. Noguera V. (2002). Physico-chemical and chemical properties of some coconut coconut coir dusts for use as a peat substitute for containerised ornamental crops. *Bioresour. Technol.* 82 241–245. 10.1016/S0960-8524(01)00189-4 11991072

[B2] Ao Y. Sun M. Li Y. (2008). Effect of organic substrates on available elemental contents in nutrient solution. *Bioresour. Technol.* 99 5006–5010. 10.1016/j.biortech.2007.09.011 17967534

[B3] Asaduzzaman M. Kobayashi Y. Mondal M. F. Ban T. Matsubara H. Adachi F. (2013). Growing carrots hydroponically using perlite substrates. *Sci. Hortic.* 159 113–121. 10.1016/j.scienta.2013.04.038

[B4] Barrett G. E. Alexander P. D. Robinson J. S. Bragg N. C. (2016). Achieving environmentally sustainable growing media for soilless plant cultivation systems – A review. *Sci. Hortic.* 212 220–234. 10.1016/j.scienta.2016.09.030

[B5] Bunt A. C. (1988). *Media Mixes for Container Grown Crops.* London: Unwin Yyman 10.1007/978-94-011-7904-1

[B6] Carmona E. Moreno M. T. Avilés M. Ordovás J. (2012). Use of grape marc compost as substrate for vegetable seedlings. *Sci. Hortic.* 137 69–74. 10.1016/j.scienta.2012.01.023

[B7] Cerozi B. D. S. Fitzsimmons K. (2016). The effect of pH on phosphorus availability and speciation in an aquaponics nutrient solution. *Bioresour. Technol.* 219 778–781. 10.1016/j.biortech.2016.08.079 27575336

[B8] Cheng A. Lin W. T. Huang R. (2011). Application of rock wool waste in cement-based composites. *Mater. Design* 32 636–642. 10.1016/j.matdes.2010.08.014

[B9] De Freitas S. T. Padda M. Wu Q. Park S. Mitcham E. J. (2011). Dynamic alternations in cellular and molecular components during blossom-end rot development in tomatoes expressing sCAX1, a constitutively active Ca2+/H+ antiporter from Arabidopsis. *Plant Physiol.* 156 844–855. 10.1104/pp.111.175208 21464475 PMC3177280

[B10] Fallovo C. Rouphael Y. Rea E. Battistelli A. Colla G. (2009). Nutrient solution concentration and growing season affect yield and quality of *Lactuca sativa* L. var. acephala in floating raft culture. *J. Sci. Food Agric.* 89 1682–1689. 10.1002/jsfa.3641

[B11] Gao J. F. (2006). *Experimental Guidance for Plant Physiology.* Beijing: Higher Education Press.

[B12] Gruda N. (2012). Sustainable peat alternative growing media. *Acta Hortic.* 927 973–980. 10.17660/ActaHortic.2012.927.120

[B13] Grunert O. Hernandez-Sanabria E. Vilchez-Vargas R. Jauregui R. Pieper D. H. Perneel M. (2016). Mineral and organic growing media have distinct community structure, stability and functionality in soilless culture systems. *Sci. Rep.* 6:18837 10.1038/srep18837 PMC4700413 26728128

[B14] Kabu K. Toop E. W. (1970). Influence of potassium-magnesium antagonism on tomato plant growth. *Can. J. Plant Sci.* 50 711–715. 10.4141/cjps70-132

[B15] Kader A. A. (2008). Flavor quality of fruits and vegetables. *J. Sci. Food Agric.* 88 1863–1868. 10.1002/jsfa.3293

[B16] Krucker M. Hummel R. L. Cogger C. (2010). Chrysanthemum production in composted and noncomposted organic waste substrates fertilized with nitrogen at two rates using surface and subirrigation. *HortScience* 45 1695–1701.

[B17] Kruse J. Abraham M. Amelung W. Baum C. Bol R. Kühn O. (2015). Innovative methods in soil phosphorus research: a review. *J. Plant Nutr. Soil Sci.* 178 43–88. 10.1002/jpln.201400327 PMC4497464 26167132

[B18] Kuisma E. Palonen P. Yli-Halla M. (2014). Reed canary grass straw as a substrate in soilless cultivation of strawberry. *Sci. Hortic.* 178 217–223. 10.1016/j.scienta.2014.09.002

[B19] Li H. S. (2010). *The Experiment Principle and Technique on Plant Physiology and Biochemistry.* Beijing: Higher Education Press.

[B20] Mao H. A. Xie D. T. Yang J. H. (2005). Relation of pH and cation saturation of orange orchard soil in Chongqing Jiangjin. *Chin. J. Soil Sci.* 36 877–879.

[B21] Mazuela P. (2005). Vegetable waste compost as substrate for melon. *Commun. Soil Sci. Plant Anal.* 36 1557–1572. 10.1081/CSS-200059054

[B22] Neocleous D. Savvas D. (2015). Effect of different macronutrient cation ratios on macronutrient and water uptake by melon (*Cucumis melon*) grown in recirculating nutrient solution. *J. Plant Nutr. Soil Sci.* 178 320–332. 10.1002/jpln.201400288

[B23] Nichols M. (2013). *Coconut Coir: Sustainable Growing Media. Practical Hydroponics and Greenhouses.* Available at: http://www.hydroponics.com.au/coirsustainable-growing-media/ [accessed February 13, 2017].

[B24] Pujos A. Morard P. (1997). Effects of potassium deficiency on tomato growth and mineral nutrition at the early production stage. *Plant Soil* 189 189–196. 10.1023/A:1004263304657

[B25] Raviv M. (2013). Composts in growing media: what’s new and what’s next? *Acta Hortic.* 982 39–47. 10.17660/ActaHortic.2013.982.3

[B26] Raviv M. Lieth J. H. (2008). *Soilless Culture Theory and Practice.* Amsterdam: Elsevier Science.

[B27] Rippy J. F. M. (2005). *Factors Affecting pH Establishment and Maintenance in Peat Moss-Based Substrates.* Raleigh, NC: North Carolina State University.

[B28] Rippy J. F. M. Nelson P. V. (2007). Cation exchange capacity and base saturation variation among Alberta, Canada, Moss Peats. *HortScience* 42 349.

[B29] Rodríguez-Delfína A. Posadas A. León-Velarde C. Mares V. Quiroz R. (2012). Effect of salt and water stress on the proline and total chlorophyll content and nutrients uptake on two sweet potato cultivars grown on soilless culture. *Acta Hortic.* 947 55–62. 10.17660/ActaHortic.2012.947.4

[B30] Schmilewski G. (2008). The role of peat in assuring the quality of growing media. *Mires Peat* 3 1–8.

[B31] Schwarz D. Öztekin G. B. Tüzel Y. Brückner B. Krumbein A. (2013). Rootstocks can enhance tomato growth and quality characteristics at low potassium supply. *Sci. Hortic.* 149 70–79. 10.1016/j.scienta.2012.06.013

[B32] Sonneveld C. (1993). “Rockwool as a substrate for greenhouse crops,” in *Biotechnology in Agriculture and Forestry* ed. Bajaj Y. P. S. (Berlin: Springer) 285–312.

[B33] Steiner C. Harttung T. (2014). Biochar as a growing media additive and peat substitute. *Solid Earth* 5 995 10.5194/se-5-995-2014

[B34] Uozumi A. Ikeda H. Hiraga M. Kanno H. Nanzyo M. Nishiyama M. (2012). Tolerance to salt stress and blossom-end rot in an introgression line, IL8-3, of tomato. *Sci. Hortic.* 138 1–6. 10.1016/j.scienta.2012.01.036

[B35] Urrestarazu M. Guillén C. Mazuela P. C. Carrasco G. (2008). Wetting agent effecte on physical properties of new and reused rockwool and coconut coconut coir waste. *Sci. Hortic.* 116 104–108.

[B36] Zhang W. Xu F. Zwiazek J. J. (2015). Responses of jack pine (*Pinus banksiana*) seedlings to root zone pH and calcium. *Environ. Exp. Bot.* 111 32–41. 10.1016/j.envexpbot.2014.11.001

[B37] Zhou B. Li H. Li X. M. (2000). Comparison of analyze methods on salt contents of plant. *Arid Zone Res.* 17 35–39.

