# Supplementary material for: Comparison of Coconut Coir, Rockwool, and Peat Cultivations for Tomato Production: Nutrient Balance, Plant Growth and Fruit Quality
Source: Front Plant Sci. 2017 Aug 2;8:1327. doi: 10.3389/fpls.2017.01327 (PMC5539188; doi:10.3389/fpls.2017.01327)
Supplement: Supplementary file 1 [file Table_1.DOCX]

Supplementary Material

**Comparison of coconut coir, rockwool and peat cultivations for tomato production: Nutrient balance, plant growth and fruit quality**

**Jing Xiong ^1,2^ , Yongqiang Tian ^3^, Jingguo Wang ^1^, Wei Liu ^2*^, Qing Chen ^1*^**

*** Correspondence:** Corresponding Author: [liuwei@nercv.org](mailto:liuwei@nercv.org) (W. Liu), [qchen@cau.edu.cn](mailto:qchen@cau.edu.cn) (Q. Chen).

# Supplementary Figures and Tables

For more information on Supplementary Material and for details on the different file types accepted, please see [here](http://home.frontiersin.org/about/author-guidelines#SupplementaryMaterial).

## Supplementary Figures

Ratio between different ions


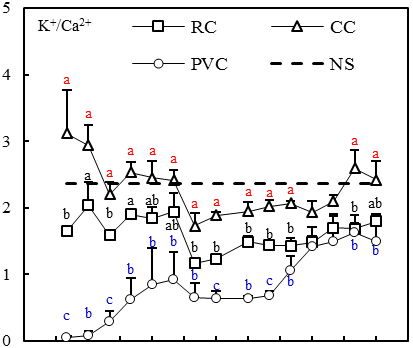

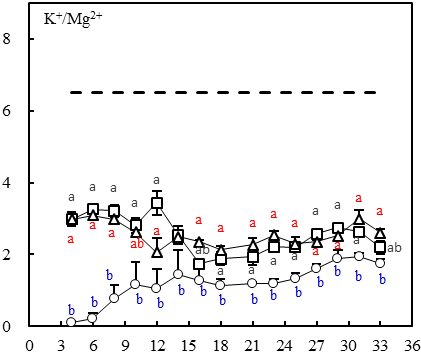

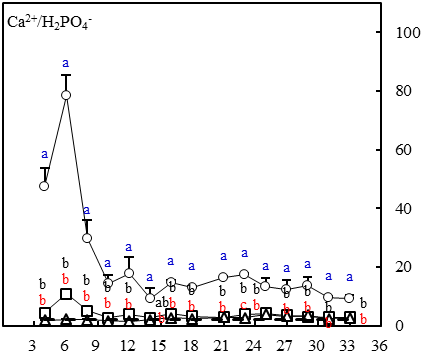


Weeks after transplanting


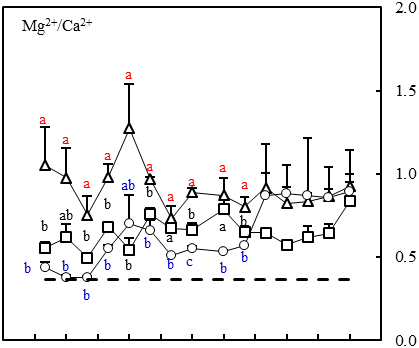

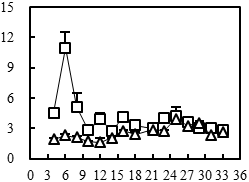


Ratio between different ions

**Fig. S1** The K^+^/Ca^2+^, Mg^2+^/Ca^2+^, K^+^/ Mg^2+^ and Ca^2+^/H_2_PO_4_^-^ ratios in root-zone solution under rockwool (RC), coir (CC) and peat-vermiculite (PVC) cultivations. denotes nutrient solution. The vertical bars represent the standard errors. Different letters indicate significant difference between treatments according LSD test at *P*<0.05. Black letter, red letter and blue letter denote rockwool (RC), coir (CC) and peat-vermiculite (PVC) cultivations respectively.


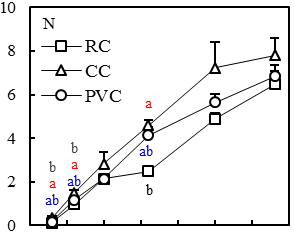

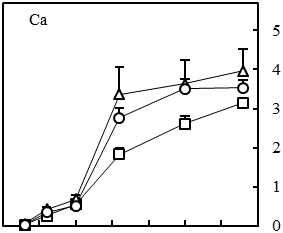

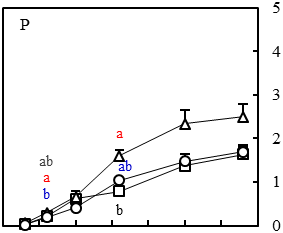

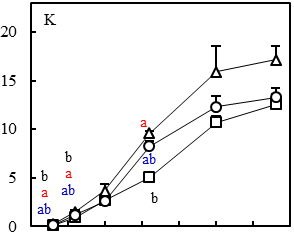

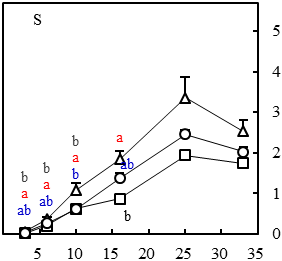

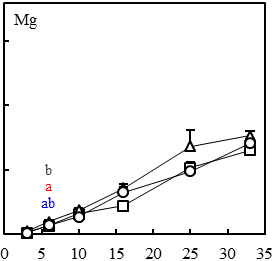


Nutrient accumulation of plant (g plant^-1^)

Weeks after transplanting

Nutrient accumulation of plant (g plant^-1^)

**Fig. S2** Nutrient uptake in crops under rockwool (RC), coir (CC) and peat-vermiculite (PVC) cultivations. The vertical bars represent the standard errors. Different letters indicate significant difference between treatments according LSD test at *P*<0.05. Black letter, red letter and blue letter denote rockwool (RC), coir (CC) and peat-vermiculite (PVC) cultivations respectively.

## Supplementary Tables

**Table S1** Malondialdehyde, antioxidative enzymes and photosynthesis in leaves under rockwool (RC), coir (CC) and peat-vermiculite (PVC) cultivations

| Substrates | Pn  (µmol CO_2_ m^-2^ s^-1^) | E  ( mmol m^-2^ s^-1^) | Gs  ( mmol m^-2^ s^-1^) | Ci  (µmol CO_2_ m^-2^ mol^-1^) |
| --- | --- | --- | --- | --- |
| RC | 1.4 b^a^ | 0.5 b | 99.0 b | 183.3 b |
| CC | 3.9 a | 1.7 a | 106.8 a | 217.5 a |
| PVC | 3.6 a | 1.7 a | 105.8 a | 215.8 a |
| Substrates | MDA  (mmol g^-1^FW) | SOD  (µg g^-1^ FW h^-1^) | POD  (µg g^-1^ FW min^-1^) | CAT  (µg g^-1^ FW min^-1^) |
| RC | 0.3 a | 360.5 a | 71.9 a | 156.5 a |
| CC | 0.2 a | 385.7 a | 75.2 a | 159.5 a |
| PVC | 0.2 a | 448.6 a | 76.7 a | 157.2 a |

^a^ The same letter denotes no significant difference among different substrates (*P* = 0.05). Pn: net photosynthetic rate, Gs: stomatal conductance, Ci: intercellular CO_2_ concentration, E: evaporation rate, MDA: malondialdehyde, SOD: superoxide dismutase, CAT: catalase, POD: peroxidase.

**Table S2** Fruit quality under rockwool (RC), coir (CC) and peat-vermiculite (PVC) cultivations

| Truss | Treatments | Soluble solids  (%) | Organic acids  (g 100g^-1^) | Reducing sugars  (%) | Vitamin C  (mg 100g^-1^) |
| --- | --- | --- | --- | --- | --- |
| 1st Truss | RC | 6.2 a^a^ | 0.38 b | 1.4 a | 55.8 a |
|  | CC | 6.4 a | 0.54 a | 1.4 a | 65.2 a |
|  | PVC | 6.1 a | 0.34 b | 1.2 a | 67.6 a |
| 5th Truss | RC | 7.9 a | 0.04 a | 3.5 a | 144.4 a |
|  | CC | 7.4 a | 0.03 a | 3.2 a | 136.2 a |
|  | PVC | 8.2 a | 0.04 a | 3.8 a | 152.1 a |

^a^ The same letter denotes no significant difference among different substrates (*P* = 0.05).
